# Impact of Different Serum Potassium Levels on Postresuscitation Heart Function and Hemodynamics in Patients with Nontraumatic Out-of-Hospital Cardiac Arrest

**DOI:** 10.1155/2018/5825929

**Published:** 2018-04-05

**Authors:** Yan-Ren Lin, Yuan-Jhen Syue, Tsung-Han Lee, Chu-Chung Chou, Chin-Fu Chang, Chao-Jui Li

**Affiliations:** ^1^Department of Emergency Medicine, Changhua Christian Hospital, Changhua, Taiwan; ^2^School of Medicine, Kaohsiung Medical University, Kaohsiung, Taiwan; ^3^School of Medicine, Chung Shan Medical University, Taichung, Taiwan; ^4^Department of Anaesthesiology, Kaohsiung Chang Gung Memorial Hospital, Chang Gung University College of Medicine, Kaohsiung, Taiwan; ^5^Department of Emergency Medicine, Kaohsiung Chang Gung Memorial Hospital, Chang Gung University College of Medicine, Kaohsiung, Taiwan; ^6^Department of Public Health, College of Health Science, Kaohsiung Medical University, Kaohsiung, Taiwan

## Abstract

**Background:**

Sustained return of spontaneous circulation (ROSC) can be initially established in patients with out-of-hospital cardiac arrest (OHCA); however, the early postresuscitation hemodynamics can still be impaired by high levels of serum potassium (hyperkalemia). The impact of different potassium levels on early postresuscitation heart function has remained unclear. We aim to analyze the relationship between different levels of serum potassium and postresuscitation heart function during the early postresuscitation period (the first hour after achieving sustained ROSC).

**Methods:**

Information on 479 nontraumatic OHCA patients with sustained ROSC was retrospectively obtained. Measures of early postresuscitation heart function (rate, blood pressure, and rhythm), hemodynamics (urine output and blood pH), and the duration of survival were analyzed in the case of different serum potassium levels (low: <3.5; normal: 3.5–5; high: >5 mmol/L).

**Results:**

Most patients (59.9%, n = 287) had previously presented with high levels of potassium. Bradycardia, nonsinus rhythm, urine output <1 ml/kg/hr, and acidosis (pH < 7.35) were more common in patients with high levels of potassium (all *p* < 0.05). Compared with hyperkalemia, a normal potassium level was more likely to be associated with a normal heart rate (OR: 2.97, 95% CI: 1.74–5.08) and sinus rhythm (OR: 2.28, 95% CI: 1.45–3.58). A low level of potassium was more likely to be associated with tachycardia (OR: 3.54, 95% CI: 1.32–9.51), urine output >1 ml/kg/hr (OR: 5.35, 95% CI: 2.58–11.10), and nonacidosis (blood pH >7.35, OR: 7.74, 95% CI: 3.78–15.58). The duration of survival was shorter in patients with hyperkalemia than that in patients whose potassium levels were low or normal (*p* < 0.05).

**Conclusion:**

Early postresuscitation heart function and hemodynamics were associated with the serum potassium level. A high potassium level was more likely to be associated with bradycardia, nonsinus rhythm, urine output <1 ml/kg/hr, and acidosis. More importantly, a high potassium level decreased the duration of survival.

## 1. Introduction

The average survival rate of patients with out-of-hospital cardiac arrest (OHCA) is only 3 to 15% [1–6]. Although a few patients can be successfully resuscitated by cardiopulmonary resuscitation (CPR) in the emergency department (ED), up to only 39% of them ultimately survive to discharge [5, 7, 8]. Some previous studies reported that the potential predictors of outcomes were early prehospital resuscitation, high-quality CPR, early percutaneous coronary intervention, and restoration of electrolyte balance [5, 9–15]. Among these factors, the serum potassium level was recognized as a key factor reflecting certain current body conditions, including abnormal cellular metabolism, the degree of cell death, electrolyte exchange, and insufficient kidney function [16–19]. Clinically, a high level of serum potassium (hyperkalemia) might potentially result in life-threatening complications by changing the action potential of heart rhythm cells and further inducing critical cardiac arrhythmia. Once cardiac arrhythmia has occurred, the impact on hemodynamic status is substantial. Cardiac output and vital organ perfusion can both collapse in a short period of time [16, 20–22].

The heart cells of OHCA patients unavoidably suffer hypoxic injury or ischemia/reperfusion injury during the cardiac arrest period (i.e., a period of no or low perfusion) [23–25]. The resting membrane potential of these damaged heart cells is more easily affected than that of normal heart cells [26–29]. Since a high level of serum potassium changes the resting membrane potential and decreases the ability to reach the action potential, we suspect that the impact of hyperkalemia on the regulation of the heart rhythm would be more obvious during the early postresuscitation period (the first hour after achieving sustained return of spontaneous circulation (ROSC)). We also suspect that the postresuscitation heart function and hemodynamic status (i.e., blood pressure, heart rate, heart rhythm, and the amount of urine output) established by CPR might be more easily destroyed by hyperkalemia [30, 31].

However, the relationship between different levels of serum potassium and the outcomes of OHCA patients had not been well addressed. In particular, the impact on early postresuscitation heart function was still unclear. Therefore, in this study, we aimed to analyze the relationships between different serum potassium levels and measures of postresuscitation heart function during the early postresuscitation period.

## 2. Methods

### 2.1. Study Design

Adult patients presenting with nontraumatic OHCA at the EDs of the Changhua Christian Hospital (CCH) medical system (one medical center and eight satellite hospitals) from January 1, 2009, to December 31, 2012, were included in this study. Patient characteristics, demographics, and serum potassium levels (low, normal, and high) that might correlate with postresuscitation heart function and hemodynamics were analyzed.

### 2.2. Ethics Statement

This paper reports a retrospective study. The protocol of this study was approved by the Institutional Review Board (IRB) of Changhua Christian Hospital (permission code: 121007).

### 2.3. Study Setting and Population

The medical records of patients presenting with nontraumatic OHCA at the EDs of the CCH medical system during the study period were retrospectively reviewed. The CCH medical system is located in central Taiwan and covers a population of almost 2,500,000. Electronic medical records can be shared among these hospitals. Nontraumatic OHCA patients were not included in this study if they had any one of the following characteristics: (1) cardiac arrest was caused by trauma, electric or burn injuries, or drowning; (2) age was <19 years (pediatric patients); (3) no resuscitation attempts were performed; (4) sustained ROSC was not achieved; and (5) medical records were incomplete (including a lack of laboratory data). Therefore, this study ultimately included 479 patients.

### 2.4. Study Protocol

Information regarding the prehospital resuscitation (including the period from scene to hospital) was obtained from public emergency medical system records or witness statements. Information on patient characteristics, demographics, clinical features, postresuscitation hemodynamics, outcome, and survival was obtained from medical charts (records made by physicians or nurses). All data were recorded according to the Utstein report system [32]. The resuscitation attempts for each patient in the EDs were based on advanced cardiopulmonary life support (ACLS), which is a standard resuscitation protocol supported by the American Heart Association (AHA). In this study, the possible etiologies that caused OHCA were classified as follows: (1) infection; (2) cardiovascular disease; (3) malignancy; (4) asphyxia; (5) electrolyte problem; (6) hypovolemia; and (7) other or unknown cause. The initial cardiac rhythm upon presentation to the ED was obtained using an electrocardiographic monitor. The rhythms were classified as asystole, pulseless electrical activity (PEA) or ventricular fibrillation (VF). In this study, VF also included pulseless ventricular tachycardia. The rhythm was obtained immediately upon arrival of the patient. In addition, inhospital CPR duration was defined as the CPR time in the ED. Once a patient achieved a sustained ROSC, the time after sustained ROSC was defined as the postresuscitation period. The initial laboratory data (the median data on blood gas, potassium levels, and creatinine clearance measured in the first 24 hours of the postresuscitation period) that might correlate with the chance of survival to discharge were analyzed.

The initial postresuscitation heart function measures and hemodynamics were recorded in the first hour of the postresuscitation period. Heart function-related factors were heart rate (tachycardia, normal, and bradycardia), blood pressure (hypertension, normal, and hypotension), and heart rhythm (sinus and nonsinus rhythms). The factor used to reflect kidney function and end organ perfusion was urine output (>1, <1 ml/kg/hr). The final factor used to reflect cell metabolism or gas exchange was blood pH level, which was recorded as acidosis (pH < 7.35) or nonacidosis (pH > 7.35). Since the values of these measurements can be very dynamic, the values were classified according to the mean value if more than one value was recorded during the first hour of the postresuscitation period. Finally, the associations between the above factors and different serum potassium levels (low: <3.5; normal: 3.5–5; high: >5 mmol/L) were analyzed. To understand the detailed dynamic changes in heart rates, the mean values of postresuscitation heart rates were recorded according to four different time periods (up to 15, 16–30, 31–45, and 46–60 minutes). The correlation between the mean heart rates and different potassium levels was analyzed.

### 2.5. Data Analysis

Descriptive analyses were reported as percentage or mean ± standard deviation (SD) for independent variables (including age, sex, possible etiology, the period from the scene to the hospital, initial cardiac rhythm, inhospital CPR duration, and outcome). The associations between postresuscitation, heart function measures, and hemodynamics and different serum potassium levels were analyzed using the chi-square test. Furthermore, we used multinomial logistic regression analysis to determine the strongest effects at different potassium levels. A correlation analysis (Spearman's rank correlation test) between prehospital resuscitation duration and different serum potassium levels was performed. Laboratory data were also compared between survivors and nonsurvivors using the Mann–Whitney *U* test. The mean dynamic heart rates in patients with different potassium levels were analyzed by using one-way ANOVA. Finally, the relationships between the duration of survival and different potassium levels were analyzed using survival analyses (Kaplan–Meier curves).

## 3. Results

### 3.1. Patient Characteristics and Demographics

During the study period, a total of 479 patients were included. Their characteristics are shown in Table 1. Most of the patients presented with asystole (62.4%, *n*=299) as their initial cardiac rhythm when arriving at the ED. Of all the patients, 25.3% (*n*=132) survived to discharge from the hospital.

### 3.2. Serum Potassium Levels Influenced Hemodynamics

The associations between initial postresuscitation hemodynamics, heart function measures, and serum potassium levels are presented in Table 2. The heart rate, heart rhythm, urine output, and blood pH level differed significantly between the three levels of serum potassium (all *p* < 0.05). Comparing to the patients with low or normal levels of potassium, the patients with high potassium levels (hyperkalemia) were significantly more likely to suffer bradycardia (41.1%, *n*=118), nonsinus rhythm (46.0%, *n*=132), urine output < 1 ml/kg/hr (69.3%, *n*=199), and acidosis (87.8%, *n*=252). Finally, we also found that the longer prehospital resuscitation duration significantly associated with the higher levels of potassium (*r*=0.75,  *p* < 0.05).

### 3.3. Considering the Strength of Effects

Multinomial logistic regression analysis was performed to consider the strength of the effects on heart function and hemodynamic status (Table 3). Compared with a high level of potassium, a normal level was associated with a higher likelihood of presenting with a normal heart rate (OR: 2.97, 95% CI: 1.74–5.08) and sinus rhythm (OR: 2.28, 95% CI: 1.45–3.58). In addition, a low level of potassium was associated with a higher likelihood of presenting with tachycardia (OR: 3.54, 95% CI: 1.32–9.51), urine output > 1 ml/kg/hr (OR: 5.35, 95% CI: 2.58–11.10), and nonacidosis (blood pH > 7.35, OR: 7.74, 95% CI: 3.78–15.58).

### 3.4. Dynamic Heart Rates

**Figure 1:**
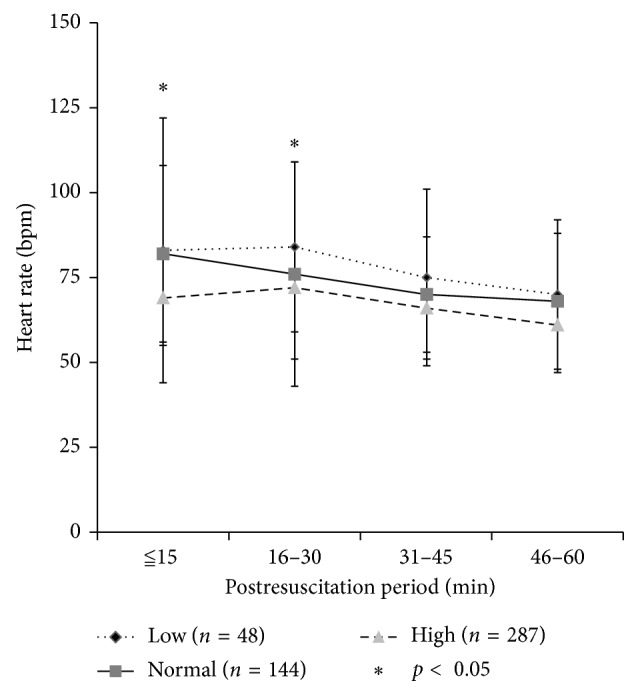
The dynamic postresuscitation heart rates at different serum potassium levels.

The dynamic postresuscitation heart rates at different serum potassium levels are shown in Figure 1. Compared with low or normal potassium levels, a high level was clearly related to slower mean heart rates, especially in the first 30 minutes of the postresuscitation period (both *p* < 0.05).

### 3.5. Potassium Levels in Serum Were Associated with Survival

**Figure 2:**
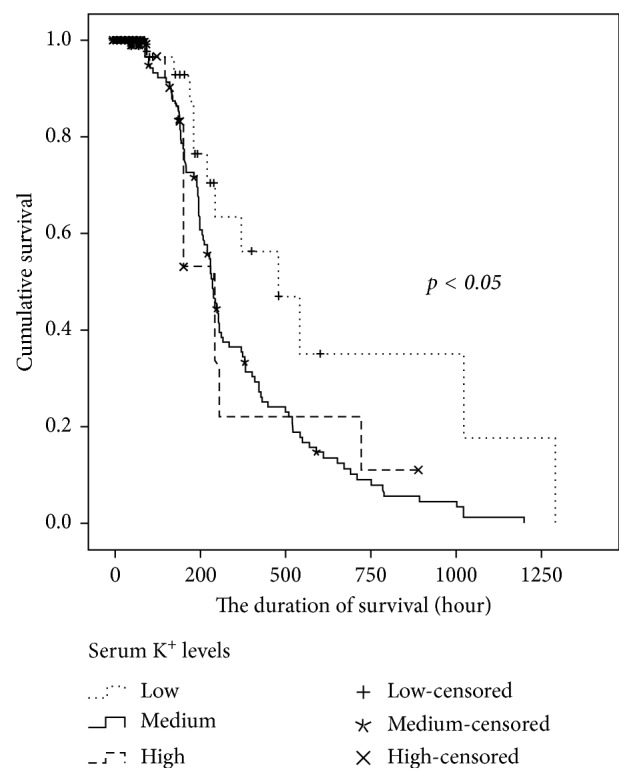
Time-related survival analysis. The serum potassium levels significantly influenced the duration of survival. The patients with high levels of potassium had the shortest duration of survival.

Initial laboratory data (including blood gas levels, pH, and potassium levels in the first 24 hours of the postresuscitation period) were significantly associated with the chance of achieving survival to discharge (all *p* < 0.05). We found that the initial potassium levels of the patients who ultimately survived to discharge were clearly lower than those of the patients who died during the hospital stay. In addition, the survivors had higher levels of pH and PaO_2_ than nonsurvivors. Initial creatinine clearance was also associated with survival to discharge (all *p* < 0.001, Table 4). Furthermore, survival analysis also showed that the patients with high levels of potassium had the shortest duration of survival (Figure 2).

## 4. Discussion

In this study, we found that a high level of serum potassium was a very common complication (59.9%, *n*=287) during the postresuscitation period. Once OHCA occurred, several factors including hypoxic injury, ischemia/reperfusion injury, cell injury, and renal failure contribute to the rise in the potassium level [16, 17, 33]. Some previous studies have demonstrated that high level potassium might induce cardiac arrest and decrease the chance of successful CPR [34, 35]. However, only few studies have focused on OHCA patients. The OHCA patients with underlying kidney dysfunction were reported to be at a higher risk of hyperkalemia than patients without kidney disease. Moreover, kidney dysfunction might not be associated with survival [11, 36]. However, the impact of hyperkalemia on heart function was not further addressed. In this study, we aim to explore this association.

The postresuscitation heart rate was clearly decreased by a high potassium level. The action potential of heart rhythm cells has been clearly demonstrated to be more difficult to reach when the potassium level is high (a high level of extracellular potassium decreased the resting membrane potential and induced cardiac arrhythmia) [16, 20, 22, 37]. Since hypoxic injury and ischemia/reperfusion injury in cardiac arrest might include heart cell injury, we suspect that the rate and rhythm of the damaged heart cells in the postresuscitation period might be more easily affected than those under normal conditions. However, this suspicion had never been clearly addressed. In this study, we found that bradycardia was more common in patients with high potassium (41.4%) levels than that in patients with low (16.7%) or normal (18.0%) potassium levels. Comparing with low or normal potassium levels, a high level of potassium was clearly related to slower mean heart rates, especially in the first 30 minutes of the postresuscitation period. After 30 minutes, the differences of heart rates presenting between low/normal/high levels were not significant (time points 31–45 and 46–60 minutes). This finding indicated that potassium levels might obviously influence the dynamic change of heart rate, especially in the first 30 minutes. Therefore, close observation and aggressive treatment for preventing hyperkalemia-related cardiac arrhythmia should be emphasized in this period.

Urine output was significantly lower in patients with high potassium levels. Some previous studies reported that decreased kidney function was one of the most common causes of hyperkalemia (because potassium excretion was decreased) [16, 38, 39]. Moreover, the amount of urine output is clinically used to reflect the condition of glomerular filtration rate and tubular flow, which are important characteristics of decreased kidney function [40–42]. Therefore, close monitoring of the urine output in the early postresuscitation period might help ED physicians to recognize kidney dysfunction and consider the possibility of hyperkalemia. Metabolic acidosis, which is associated with an increased anion gap (infection, intoxication, and lactic acidosis), was also reported to be a common cause of hyperkalemia [40, 43–45]. The increased hydrogen ions in the cells might displace potassium from the cells (resulting in an increased serum potassium level) [46–48]. We suspect that routinely checking the blood pH during the early postresuscitation period would benefit evaluation of the potassium level. Finally, patients with normal or low potassium levels are likely to present with more stable postresuscitation function and hemodynamic status. Logistic regression analysis showed that patients with low levels of potassium were more likely to present with tachycardia, urine output > 1 ml/kg/hr, and nonacidosis than patients with normal levels of potassium. A possible reason could be massive fluid resuscitation. Increased intravascular fluid provided kidney perfusion and balanced the pH.

Overall, we found that a higher level of serum potassium predicted worse outcomes in OHCA patients. The initial potassium level was clearly lower in survivors than that in patients who died during the hospital stay. The duration of survival was also influenced by potassium levels. Therefore, early treatment for hyperkalemia might be emphasized for patients entering the postresuscitation period.

### 4.1. Limitations

This retrospective study had some limitations. First, the association between hypothermia therapy and potassium levels was not evaluated in this study (not routinely used in each patient). Second, this study focused only on the early postresuscitation period (the first hour), whereas a high level of potassium might cause persistent complications. Finally, the patient outcomes in this study did not include neurological conditions (patient number is not enough to provide reliable evidence).

## 5. Conclusion

The impact of different potassium levels on early postresuscitation heart function was not clear until this study has more clearly stated. Early postresuscitation heart function and hemodynamics were associated with the serum potassium level. A high potassium level was more likely to be associated with bradycardia, nonsinus rhythm, urine output <1 (ml/kg/hr), and acidosis. The association between potassium levels and neurological outcomes should be analyzed by more patient number and analysis in the future. Most importantly, a high level of potassium decreased the duration of survival.

## Figures and Tables

**Table 1:** Characteristics and clinical features of the patients.

	Patients who suffered nontraumatic OHCA and achieved sustained ROSC (*n*=479)	
	Number	%
Personal information		
Age (mean ± SD) (y/o)	70.7 ± 12.4	
Male	271	56.6
Possible etiologies		
Infection	99	20.7
Cardiovascular disease	143	29.9
Malignancy	45	9.4
Asphyxia	114	23.8
Electrolyte problem	13	2.7
Hypovolemia	29	6.1
Other or unknown cause	36	7.5
Prehospital information		
Period from scene to hospital (mean ± SD) (min)	24.8 ± 10.8	
Inhospital resuscitation		
Initial cardiac rhythm		
Asystole	299	62.4
PEA	70	14.6
VF^∗^	110	23.0
Inhospital CPR duration (mean ± SD) (min)	16.9 ± 9.4	
Outcome measurement		
Survival to discharge	132	25.3

^∗^VF includes patients with pulseless VT.

**Table 2:** Serum potassium levels influence the initial postresuscitation heart function and hemodynamic status.

	Total patients (*n*=479)	Serum potassium level			*p* value
		Low (K^+^ < 3.5, *n*=48)	Normal (K^+^ = 3.5–5, *n*=144)	High (K^+^ > 5, *n*=287)	
	Number (%)	Number (%)	Number (%)	Number (%)	
Heart rate^∗^					<0.001
Tachycardia	121 (25.3)	17 (35.4)	43 (29.9)	61 (21.3)	
Normal	206 (43.0)	23 (47.9)	75 (52.1)	108 (37.6)	
Bradycardia	152 (31.7)	8 (16.7)	26 (18.0)	118 (41.1)	
Blood pressure					0.353
Hypertension	149 (31.1)	11 (22.9)	49 (34.0)	89 (31.0)	
Normal	142 (29.6)	16 (33.3)	47 (32.6)	79 (27.5)	
Hypotension	188 (39.3)	21 (43.8)	48 (33.4)	119 (41.5)	
Heart rhythm^∗^					0.001
Sinus rhythm	291 (60.8)	33 (68.8)	103 (71.5)	155 (54.0)	
Nonsinus rhythm	188 (39.2)	15 (31.2)	41 (28.5)	132 (46.0)	
Urine output (median)^∗^					<0.001
>1 (ml/kg/hr)	174 (36.3)	35 (72.9)	51 (35.4)	88 (30.7)	
<1 (ml/kg/hr)	305 (63.7)	13 (27.1)	93 (64.6)	199 (69.3)	
Blood pH^∗^					
Acidosis (<7.35)	374 (78.1)	20 (41.7)	102 (70.8)	252 (87.8)	<0.001
Nonacidosis (>7.35)	105 (21.9)	28 (58.3)	42 (29.2)	35 (12.2)	

^∗^Significant factors; the serum K^+^ level is given in units of mmol/L.

**Table 3:** Multinomial logistic regression analysis for analyzing the strength of effects on heart function and hemodynamic status at different serum potassium levels.

	Serum potassium level				
	Low (K^+^ < 3.5)		Normal (K^+^ = 3.5–5)		High^†^ (K^+^ > 5)
	OR	95% CI	OR	95% CI	—
Heart rate					
Tachycardia^∗^	3.54	1.32–9.51	2.85	1.56–5.23	1
Normal^∗^	2.68	1.05–6.82	2.97	1.74–5.08	1
Bradycardia^†^	—	—	—	—	—
Blood pressure					
Hypertension	0.47	0.19–1.12	1.05	0.62–1.76	1
Normal	1.01	0.45–2.28	1.33	0.79–2.26	1
Hypotension^†^	—	—	—	—	—
Heart rhythm					
Sinus rhythm^∗^	2.05	0.99–4.24	2.28	1.45–3.58	1
Nonsinus rhythm^†^	—	—	—	—	—
Urine output (median)					
>1 (ml/kg/hr)^∗^	5.35	2.58–11.1	1.17	0.75–1.85	1
<1 (ml/kg/hr)^†^	—	—	—	—	—
Blood pH					
Nonacidosis (>7.35)^∗^	7.74	3.78–15.85	2.67	1.57–4.53	1
Acidosis (<7.35)^†^	—	—	—	—	—

^†^Reference group; ^∗^significant factors; OR: odds ratio; CI: confidence interval; the serum K^+^ level is given in units of mmol/L.

**Table 4:** Initial potassium levels in serum were associated with the chance of survival (the median laboratory data during the first 24 hours of the postresuscitation period).

	Total patients (*n*=479)	Survival to discharge (*n*=132)		
		Success	Failure	*p* value
Initial K level (median) (mmol/L)	4.4	4.2	5.1	<0.001
Initial pH (median)	7.10	7.14	7.08	<0.001
Initial PaO_2_ level (median) (mmHg)	164.2	185.2	127.3	<0.001
Initial PaCO_2_ level (median) (mmHg)	58.6	49.0	62.7	<0.001
Initial creatinine clearance (median) (mL/s)	1.2	0.9	1.4	<0.001
